# Degradation of a textile reactive azo dye by a combined biological-photocatalytic process: *Candida tropicalis* Jks2 -Tio_2_/Uv

**DOI:** 10.1186/1735-2746-9-33

**Published:** 2012-12-23

**Authors:** Narjes Jafari, Rouha Kasra-Kermanshahi, Mohammad Reza Soudi, Amir Hossein Mahvi, Sara Gharavi

**Affiliations:** 1 Department of Biology, Faculty of Science, Alzahra University, Tehran, Iran; 2 School of Public Health, Tehran University of Medical Sciences, Tehran, Iran

**Keywords:** Azo dyes, *Candida tropicalis*, Combined wastewater treatment

## Abstract

In the present study, the decolorization and degradation of Reactive Black 5 (RB5) azo dye was investigated by biological, photocatalytic (UV/TiO_2_) and combined processes. Application of *Candida tropicalis* JKS2 in treatment of the synthetic medium containing RB5 indicated complete decolorization of the dye with 200 mg/L in less than 24 h. Degradation of the aromatic rings, resulting from the destruction of the dye, did not occur during the biological treatment. Mineralization of 50 mg/L RB5 solution was obtained after 80 min by photocatalytic process (in presence of 0.2 g/L TiO_2_). COD (chemical oxygen demand) was not detectable after complete decolorization of 50 mg/L RB5 solution. However, photocatalytic process was not effective in the removal of the dye at high concentrations (≥200 mg/L). With 200 mg/L concentration, 74.9% of decolorization was achieved after 4 h illumination under photocatalytic process and the absorbance peak in UV region (attributed to aromatic rings) was not completely removed. A two-step treatment process, namely, biological treatment by yeast followed by photocatalytic degradation, was also assessed. In the combined process (with 200 mg/L RB5), absorbance peak in UV region significantly disappeared after 2 h illumination and about 60% COD removal was achieved in the biological step. It is suggested that the combined process is more effective than the biological and photocatalytic treatments in the remediation of aromatic rings.

## Introduction

Azo dyes, representing one group of common textile dyes, contain one to four azo groups usually attached to two radicals of which at least one, but usually both, is aromatic groups. These molecules are chemically stable and difficult to biodegrade aerobically [1]. More than 100,000 commercial dyes are available to textile industries with over 700,000 tons of commercial dye being produced annually. The reactive azo dyes are highly recalcitrant to conventional wastewater treatment processes. In fact, as much as 90% of reactive dyes can remain unaffected after activated sludge treatment [2]. An estimated 10-15% of the synthetic dyes are discharged into wastewater as effluent which carries the potential to cause environmental damage [3]. In many cases, colored textile wastewater is aesthetically unacceptable, hinders light penetration, damages the quality of the receiving streams, and may be inhibitory towards biological wastewater treatment systems, to food chain organisms and to aquatic life. Consequently, it is important to remove dyestuff from the wastewater before discharging it to the environment [4]. The strong electron-withdrawing groups characterizing the chemical structure of dyes protect them from the bacterial oxygenases [5]. Therefore, conventional activated sludge systems have difficulty in handling such wastewater, particularly in removing the color [4]. The traditional treatment techniques applied in textile wastewater, such as chemical coagulation/flocculation, membrane separation (ultrafiltration, reverse osmosis) or activated carbon adsorption are known to be costly and only transfer the pollutants from the liquid to the solid phase [4,6]. Therefore, more efficient methods for treatment of dyed effluents should be examined.

Most azo dye degrading microorganisms cleave the azo bond(s) of the respective azo dye and colorless, possibly toxic, aromatic amines are formed [7]. Thus, an alternative method must be considered for oxidization of the toxic aromatic amines.

Advanced oxidation processes (AOPs) produce a highly reactive non-specific oxidant called hydroxyl radicals (OH^·^), capable of destroying a wide range of organic pollutants in water and wastewater [2,8]; presently, destruction of organic compounds using TiO_2_/UV photocatalytic process is one of the various advanced oxidation processes applied.

Numerous advanced treatment technologies have been developed to reduce contaminants from textile wastewater. To effectively treat such wastewater, often a combined chemical and biological oxidation process is required as purely biological treatment is usually very slow or not possible at all [4].

Alternatively, recent reports (from 1999) indicate that few ascomycetous yeast species such as *Candida zeylanoides*[9,10], *C.tropicalis*, *Debaryomyces polymorphus*[11], *Issatchenkia occidentalis*[12], *C.oleophila*[6], *Galactomyces geotrichum*[13] and *C.albicans*[14] are capable of carrying out putative enzymatic biodegradation and decolorization of azo dyes.

The aim of the present study was to investigate the efficiency of photocatalytic process as a post-treatment stage in the removal of aromatic rings produced through biological treatment.

## Materials and methods

### Chemicals and dyes

**Figure 1:**
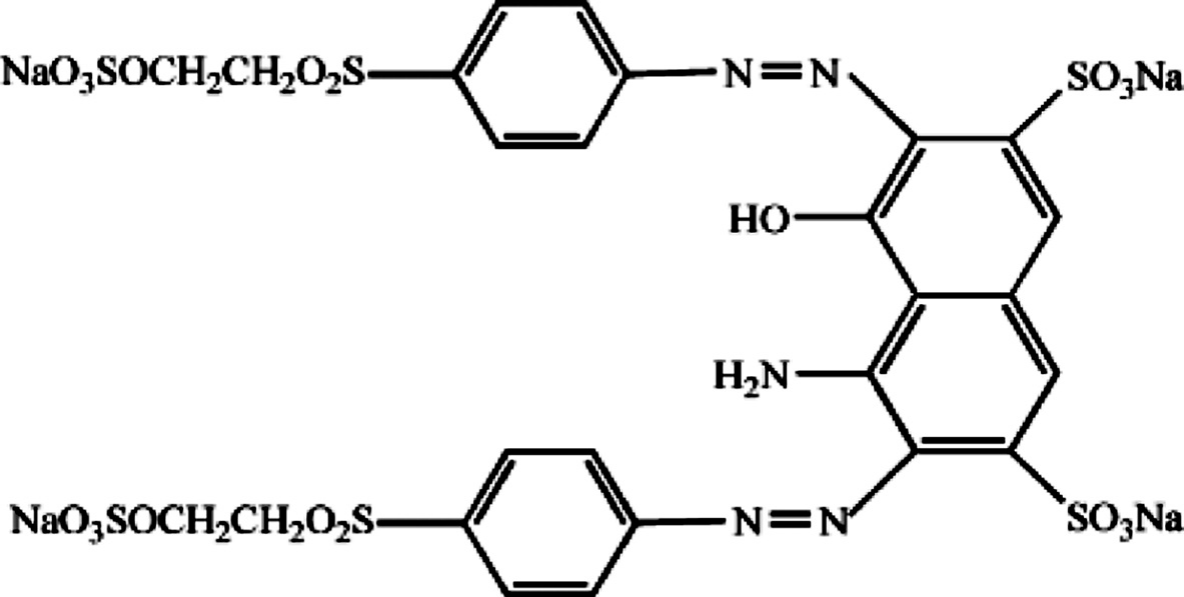
Chemical structure of RB 5.

Azo dye, Reactive Black 5 (RB5), was obtained from a local company (Alvan Sabet, Iran) and used without further purification. The dye is widely used by textile industries in Iran. Figure 1 shows the chemical structure of RB5. TiO_2_-p25 was purchased from Plasmachem GmbH company (Germany). It has a specific surface area (BET) of approximately 50 m^2^/g and an average particle size of 21 nm.

### Microorganism

The yeast strain was screened from activated sludge of municipal wastewater treatment plant and was maintained on YMA (Yeast Malt Agar containing (g/L) yeast extract (3), malt extract (3), glucose (10), peptone (5) and agar (15)) slant at 4°C. Biochemical and molecular identification tests were performed on the isolated yeast and subsequently identified as *Candida tropicalis* JKS2. The 26S rDNA partial sequence of the isolated yeast JKS2 were deposited in the GenBank database with the accession number JQ650229.

### Biological experiments and decolorization procedures

Examination of decolorization and biodegradation ability of *Candida tropicalis* JKS2 was carried out in synthetic medium containing: (glucose, 10; Yeast extract, 0.34; NH_4_Cl, 0.84; KH_2_PO_4_, 0.134; K_2_HPO_4_, 0.234; MgCl_2_.6H_2_O, 0.084) g/L and 200 mg/L RB5. The pH of the solution was adjusted to 7±0.2 before being autoclaved. A 5% (v/v) of the yeast suspension in 0.85% saline was inoculated into 100 mL of medium in 250-mL flasks and incubated in shaking incubator at 150 rpm and at 32°C for 72 h. In addition, control flasks containing the same medium without inoculums were also kept to observe the abiotic decolorization. At regular time intervals, an aliquot (4 mL) of the culture media was withdrawn from the flasks, centrifuged at 12000 rpm for 10 min and the supernatant was analyzed for the remaining dye concentration. Decolorization of dye was determined by measuring the absorbance of culture supernatant at λ_max_ (597 nm). The percentage of decolorization was calculated according to the following formulation:

(1) $$\mathrm{Percentage}\;\mathrm{of}\;\mathrm{decolorization}=\left(A_{i}-A_{t}\right)/A_{i}\times 100\;\left(1\right)$$

Where A_i_ and A_t_ are the initial absorbence of the dye solution and at sampling time (t) after inoculation, respectively [13].

Spectral scanning between 200 and 800 nm using a UV–vis spectrophotometer (Shimadzu, Japan) was performed in order to analyze the dye degradation process. In this study, absorbance measurement at 254 nm wavelength was used to indicate the presence of aromatic compound in the sample [15].

### Photocatalytic experiments

#### Photocatalytic reactor and light source

Photocatalytic experiments were performed in a batch system (pyrex vessel) with a total capacity of 1 L (batch mode). The total volume of the dye solution in the system was 800 mL. The reactor containing reaction solution (mixture of dye solution and photocatalyst powder) was placed on a magnetic stirrer to provide appropriate mixing. A medium-pressure mercury lamp (UV-C, maximum emission at 247.3 nm) with a power of 150 W and 66 mm length (manufactured by ARDA, France) was used as the light source. The UV lamp was placed centrally and parallel to the length of the cylinder in the reactor, inside a quartz tube [16,17]. The reactor was covered with an aluminum foil sheet to prevent UV emission around it. Due to the production of heat by the light source, the temperature of the solution was maintained constant at 21±3°C in all experiments by cooling water around the vessel.

#### Analytical procedures

In order to perform the photocatalytic experiments, the working solution, containing 50 mg/L of dye and 0.2 g/L of TiO_2_ was prepared using tap water. Prior to the photocatalytic process, the suspensions were magnetically stirred in the dark for 30 min to reach adsorption-desorption equilibrium between the dye and TiO_2_. Samples (5 mL) were taken at specific time intervals, centrifuged at 6000 rpm for 15 min and then filtered through a 0.45 μm syringe filter in order to separate TiO_2_ particles [18]. Measurement of absorbance was carried out at maximum wavelength of RB5 (λ_max_ =597 nm). UV–vis spectra of samples were recorded between 200 and 800 nm. Photocatalytic degradation of different concentrations of RB5, namely, 100, 200, 300 and 500 mg/L was also examined.

#### Combined biological-photochemical process

To carry out these experiments, synthetic medium was treated with *Candida tropicalis* JKS2 cells for 24 h and after centrifugation and filtration, fed into the reactor. At different time intervals, samples were withdrawn, centrifuged and filtered through a 0.45 μm membrane filter following which UV–vis scan spectroscopy and COD measurements were carried out. COD measurements (for both biological and photocatalytic steps of the process) were performed following the closed reflux, colorimetric method using a COD reactor (Hack, USA), according to standard procedures [19].

## Results

### Biological experiments

#### Candida tropicalis

JKS2 was able to remove about 85% of color in culture containing 200 mg/L RB5 after 12 h. Complete decolorization was observed in less than 24 h. The dye concentration in control flasks remained unchanged.

UV–vis spectra (200–800 nm) were studied. Reactive Black 5 presents two main absorption peaks, one in visible region (597 nm) and another in UV region (310 nm), which can be attributed to the presence of chromophoric azo bonds and both aryl and naphthalene-like moieties, respectively [6]. The results of our experiments showed that the peak at 597 nm disappeared approximately after 12 h (complete removal of this peak was observed in less than 24 h). Meanwhile the main peak at UV region (310 nm) was apparently shifted to 254 nm within the first 12 h and no more changes were observed during further incubation time up to 72 h.

#### Photocatalytic experiments

**Figure 2:**
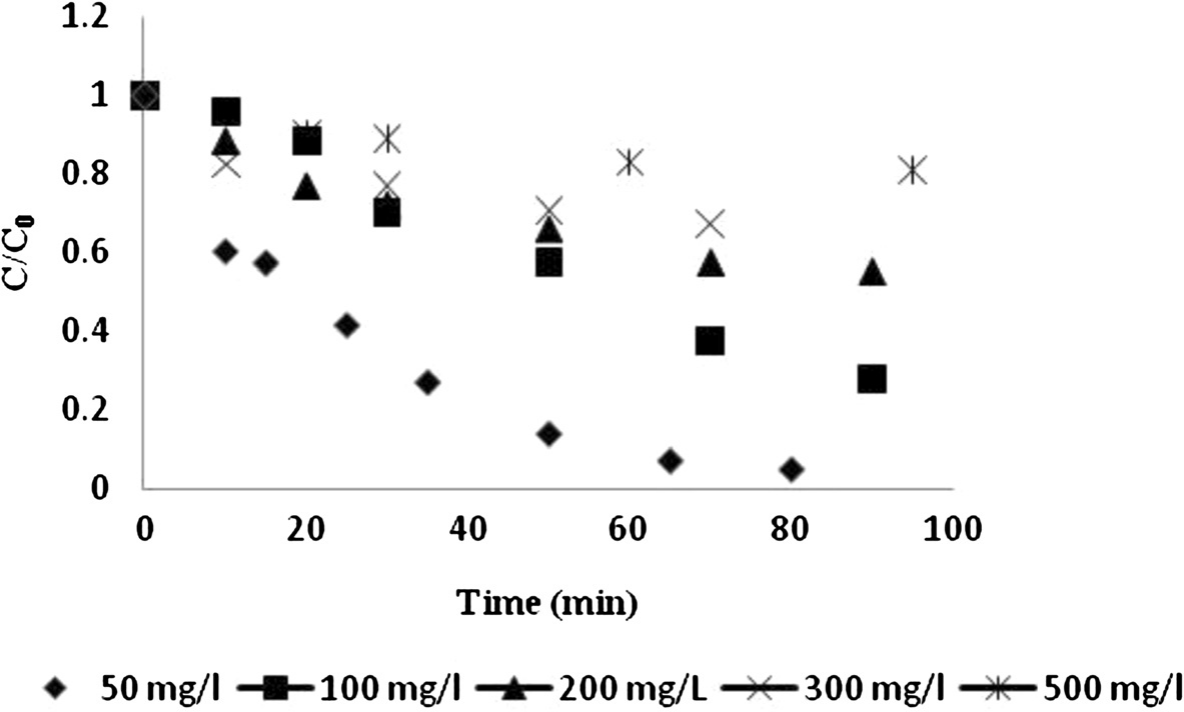
Photodegradation of RB 5 at different dye concentrations.

Photocatalytic process was employed to investigate the degradation of RB5 at 50, 100, 200, 300 and 500 mg/L concentrations. Results (Figure 2) showed that this process was effective in the removal of RB5 at low concentrations; almost complete decolorization (94.8%) was achieved within 80 min of illumination time at 50 mg/L RB5. After 90 min of exposure time, dye decolorization at 100, 200,300 and 500 mg/L RB5 concentrations was about 72.3%, 48.6%, 33.5% and 19.2%, respectively.

**Figure 3:**
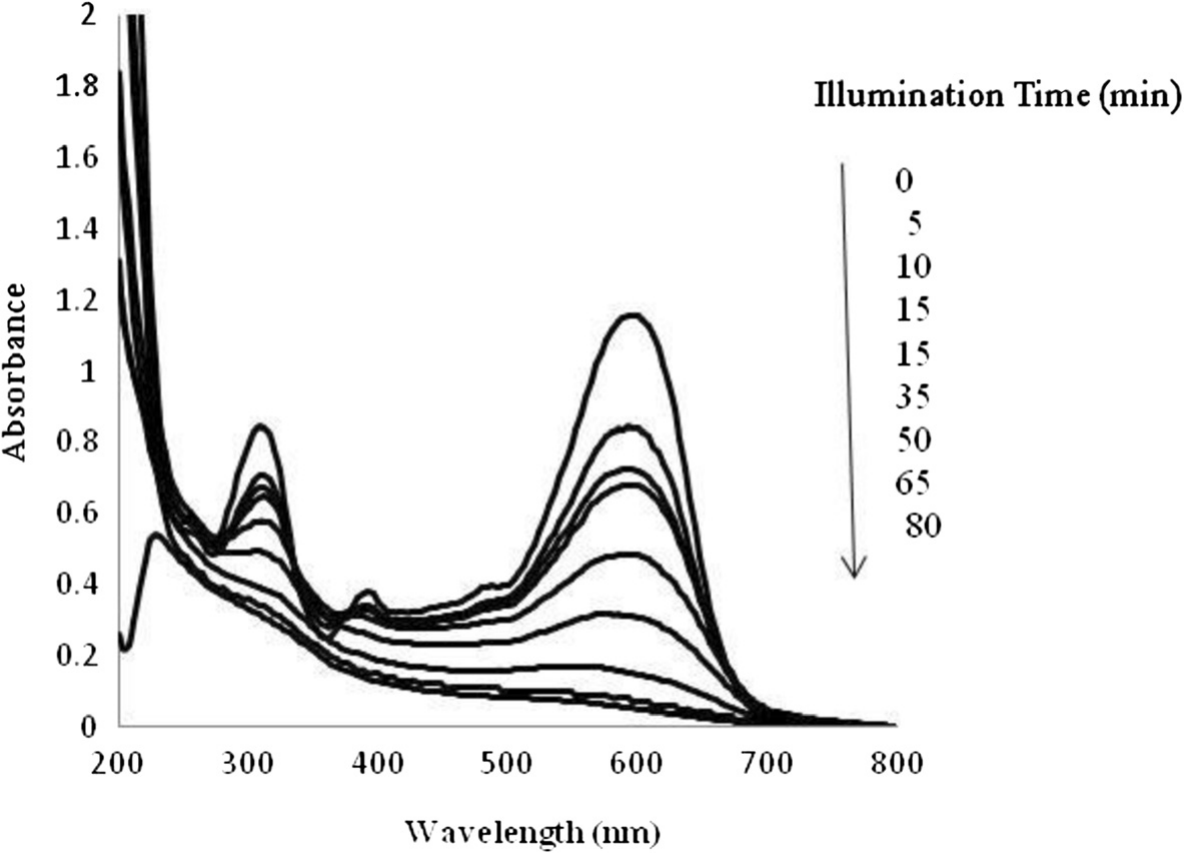
UV–vis spectra (200–800 nm) of RB 5 (50 mg/L) at different time intervals of illumination.

The UV–vis absorptive spectra of RB5 (at 50 mg/L concentration) was studied at different exposure times in the photocatalytic experiments. Results (Figure 3) showed that absorbance in the visible and UV spectral regions decreased with increasing illumination time indicating the photocatalytic oxidation was effective in removal of two absorbance peaks in visible and UV regions which belong to azo bonds and aromatic rings (benzene and naphthalene), respectively.

#### Decolorization kinetics

**Figure 4:**
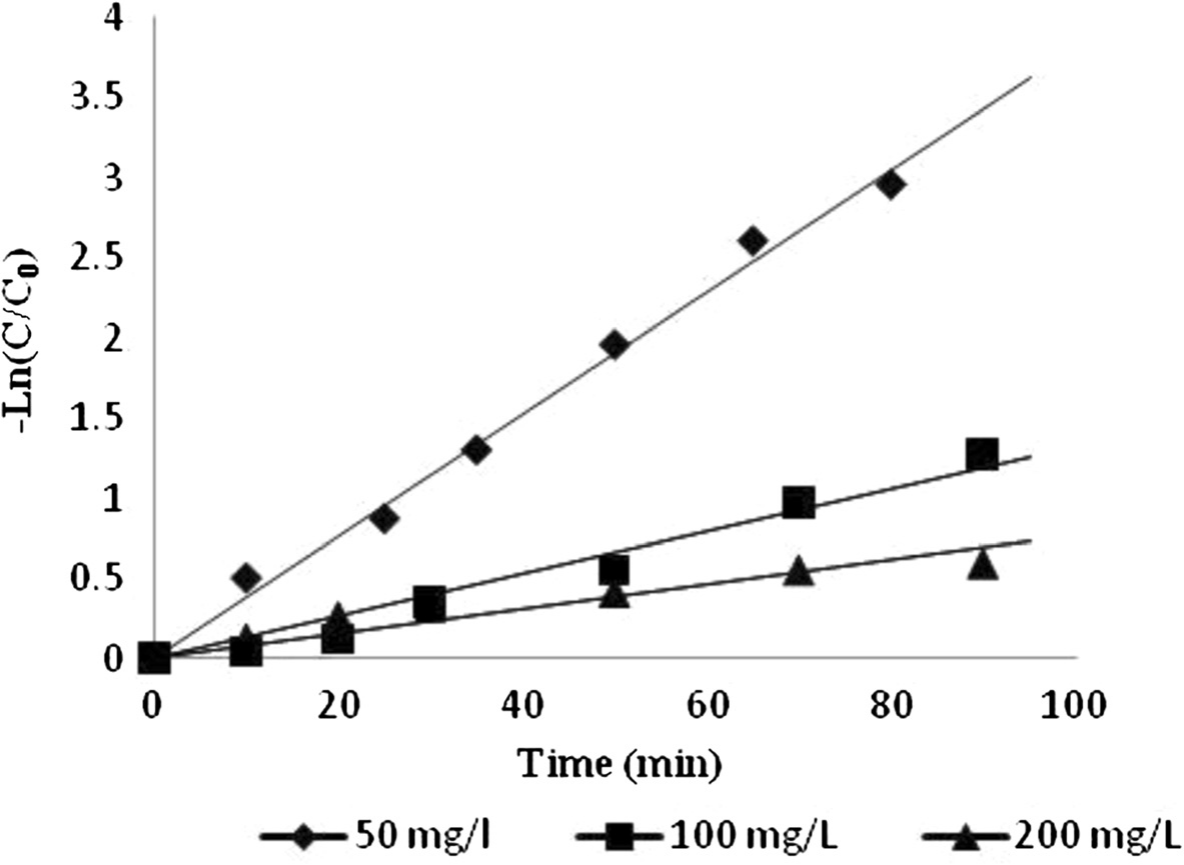
**The plot of ln C/C**_**0 **_**vs. time (at different concentrations of RB 5) indicates that the kinetic studies follow first-order reaction.**

Kinetics studies was performed for photocatalytic decolorization at 50, 100 and 200 mg/L RB5 concentrations and results (Figure 4) followed first-order kinetic model. This kinetic model was presented in equation of –Ln C/C_0_= kt, where C_0_, C, t and k are initial dye concentration, dye concentration at t (min), decolorization time (min) and decolorization rate constant (1/min), respectively [20].

Decolorization rate constant (k) was calculated from the slope of the curve. In Figure 4, the plot of Ln C/C_0_ versus t and the linear correlation between –Ln C/C_0_ and illumination time are shown. R^2^ and k values are presented in Table 1. Data showed that decolorization constant rate decreased as the initial dye concentration increased.

**Table 1:** Decolorization parameters in photocatalytic process at different concentrations of RB5

**Dye concentration (mg/l)**	**R**^2*^	**k**^♦^**(1/min)**
50	0.991	0.038
100	0.982	0.016
200	0.933	0.005

*: correlation coefficient.

♦: decolorization rate constant.

#### Combined biological-photochemical process

**Figure 5:**
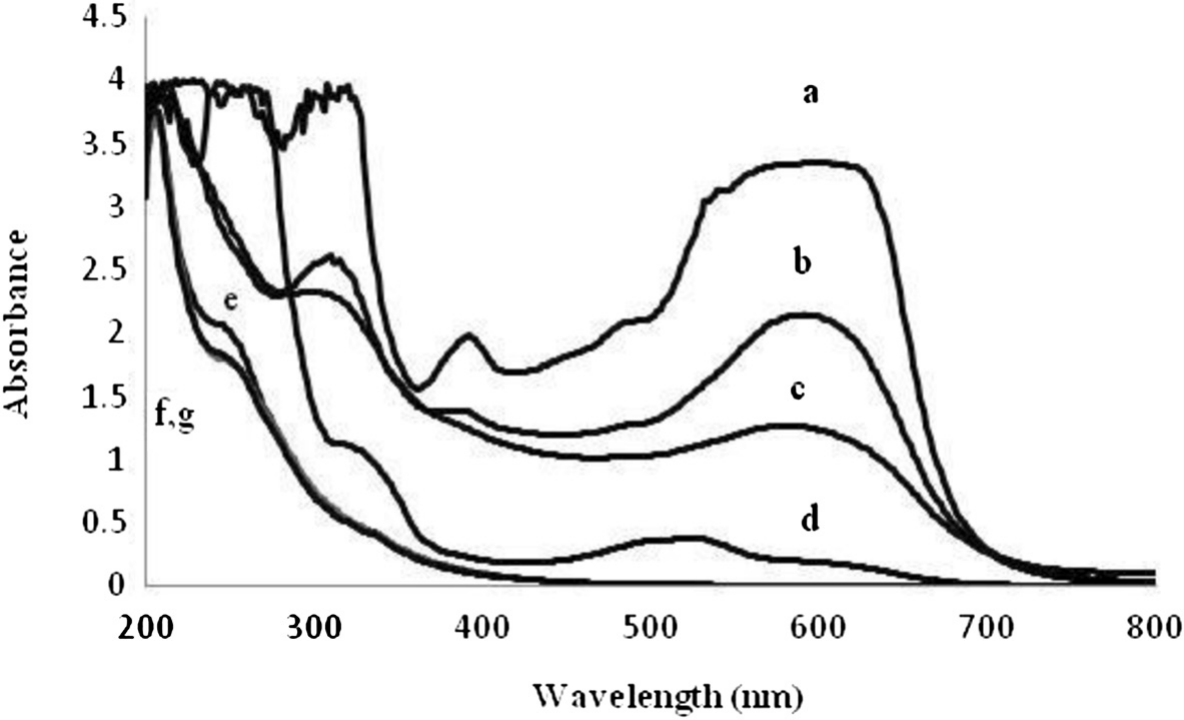
**UV–vis spectral scans, a: Control (200 mg/L RB5 without treatment), b and c: after treatment by TiO**_**2 **_**+ UV for 2 and 4 h, respectively, d: after treatment by *Candida tropicalis *JKS2 (24 h), e: after combined treatment by yeast (24 h) and TiO**_**2 **_**+ UV (2 h), f and g: after combined processing by yeast (24 h) and TiO**_**2 **_**+ UV (3 and 4 h, respectively).**

As shown in Figure 5, at 200 mg/L dye concentration, 74.9% decolorization occurred after 4 h irradiation but the peak attributed to aromatic rings in UV region did not significantly change. Results (Figure 5, curves e,f,g) showed that application of biological-photocatalytic process was more efficient than the photocalalytic and biological process solely in aromatic by-product remediation. Absorption changes were not significant between UV–vis spectra in combined process after 2 and 3 or 4 h illumination.

#### COD measurements

COD gives a measure of dye degradation and generated intermediates during the irradiation as well as a measure of the oxygen equivalent of the organic content in a sample that is susceptible to oxidation by strong oxidant [15]. In the photocatalytic process, COD was measured in both, raw and treated dye, solutions. The COD for untreated sample (at 50 mg/L RB5 concentration) was about 25 mg/L O_2_ and for treated sample was not detectable presumably due to complete mineralization of the sample. In the combined process COD measurement was performed in three stages namely, before and after biological step and at 4 points in photocatalytic step (after treatment for 1, 2, 3 and 4 h). Biological treatment was able to remove 59.9% of COD. COD reduction was not observed during photochemical post treatment.

## Discussion

Measures to develop environmentally favorable as well as cost-effective techniques to remove hazardous pollutants from textile wastewater, have received a great deal of attention.

In the present study, application of biological, photocatalytic and combined processes was investigated in the treatment of synthetic medium containing RB5. In the photocatalytic process, the amount of decolorization decreased as RB5 concentration increased (Figure 2). At a high dye concentration, a significant amount of UV may be absorbed by the dye molecules rather than the TiO_2_ particles and thus reduce the efficiency of the catalytic reaction. Another possible reason is the interference from intermediates formed upon degradation of the parental dye molecules. They may compete with the dye molecules for the limited adsorption and catalytic sites on the TiO_2_ particles and thus inhibit decolorization. Such interference would be more pronounced in the presence of an elevated level of degradation intermediates formed upon an increased initial dye concentration [16,21].

COD measurement and UV–vis spectral analysis were used as a measure of dye degradation and generated intermediates during the processes. Mineralization of 50 mg/L RB5 solution was obtained after 80 min by photocatalytic process (in presence of 0.2 g/L TiO_2_). COD value was not detectable after complete decolorization of 50 mg/L RB5 solution. However, photocatalytic process was not effective in the removal of the dye at high concentrations (≥200 mg/L). At 200 mg/L concentration, 74.9% of decolorization was achieved after 4 h illumination under photocatalytic process and the absorbance peak in UV region (attributed to aromatic rings) was not completely removed. These observations indicate the inadequacy of photocatalytic processes in removal of concentrated wastewater. Successful application of the biological treatment in decolorization of high concentrated synthetic medium (up to 1000 mg/L RB5) was noted but the remediation of aromatic rings produced from destruction of dye molecule did not occur (Figure 5), even with prolonged incubation time.

AOP, followed by biological treatment, could be justified if bio-recalcitrant compounds are easily degradable by AOP and the resulting intermediates are easily degradable by the biological treatment [22], as previously reported [4,23-29] in the degradation of biorecalcitrant organic compounds.

A few reports describe the application of photocatalytic process as a post-treatment in a combined process [22,30,31]. These investigations were suggested that since the concentration of organic compounds were high in wastewater and the use of photocatalytic process as a pre-treatment stage was not favorable. González et al. [32] evaluated the degradation of chlorophenols by sequential biological-photochemical process using *Trametes pubescens* and TiO_2_/UV. This process was suggested because partial oxidation of original molecules was occurred and intermediate molecules produced through biological process could be more toxic than original molecules [32].

In the present study, a two-step treatment process, namely, biological treatment by yeast followed by TiO_2_/UV photocatalytic degradation, was also assessed. In the combined process (at 200 mg/L RB), absorbance peak in UV region significantly disappeared after about 2 h of illumination (Figure 5) and about 60% of COD removal was achieved in the biological step but in photocatalytic step, COD reduction was not observed which could be due to the production of strongly oxidative by-products that are resistant to further oxidation by photocatalytic treatment. As previously reported by Tanaka et al. [33] further hydroxylation of aromatic intermediates products leads to the cleavage of the aromatic ring resulting in the formation of oxygen-containing aliphatic compounds [33]. It is apparent that the application of photocatalytic process for an extended period of time is not reasonable.

## Conclusion

In the present study, COD measurement and UV–vis analysis were used as a measure of degradation of dye and generated intermediates during the processes. Successful application of the biological treatment in decolorization of high concentrated synthetic medium was noted; however not in the remediation of aromatic rings produced from destruction of dye molecule. Therefore, photocatalytic process was used as a post- treatment for degradation of aromatic rings and was effective for this purpose, but COD reduction was not observed in this process which could be due to the production of by-products that are resistant to further oxidation by photocatalytic treatment. Further evaluation is required to improve the system in which three stages take place. The third step (i.e., biological treatment) subsequent to the photocatalytic step could facilitate further COD reduction. Our results showed that the combined process was more effective than biological treatment in remediation of aromatic rings (resulting from decolorization of the dye) and was cost-effective (in terms of the electrical power utilized) compared to the photocatalytic treatment only.

## Competing interests

The authors declare that they have no competing interests.

## Authors’ contributions

This manuscript was a part of the NJ^,^ s master thesis performed under RKK and MRS supervision. AHM participated in photocatalytic decolorization studies. SGH assisted in reviewing of the manuscript. All authors read and approved the final manuscript.
